# Orofacial cleft research and the Sustainable Development Goals: a bibliometric study

**DOI:** 10.7189/jogh.16.04264

**Published:** 2026-08-14

**Authors:** Anna Chiara Corriero, Felicity Vidya Mehendale

**Affiliations:** 1 NHS Lothian, Edinburgh, Scotland, UK; 2 Usher Institute, University of Edinburgh, Edinburgh, Scotland, UK

**Keywords:** Sustainable Development Goals, orofacial clefts, craniofacial surgery, congenital anomalies

## Abstract

**Background:**

Orofacial clefts (OFC) are common congenital conditions occurring in 1 of 700 births. They have lifelong nutritional, educational, and psychosocial impacts, and require multidisciplinary care from the antenatal period into adulthood. In 2010, the World Health Organization resolved to strengthen responses to congenital conditions, aligning with the United Nations Sustainable Development Goals (SDGs) 2030 targets. In this bibliometric mapping exercise based on inferred SDG themes from metadata, we evaluate the extent to which OFC research mentions these SDGs, examining differences across country income groups.

**Methods:**

We conducted a keyword search and content analysis of OFC articles published between 1842 and 2024 using a Python script developed by Elsevier. The script inferred SDG themes directly from titles, abstracts, and keywords. We analysed SDG mentions, thematic relevance, and the World Bank income classifications of publishing institutions, and used Spearman’s rank correlation to calculate the statistical significance of the correlation between SDG mentions and the income group of the country of the authors.

**Results:**

Of 39,176 publications, 5,598 (14.0%) referenced at least one SDG, yielding 6,096 unique mentions. Additionally, 4,896 (87.5%) of SDG-related OFC papers were successfully linked to an author country income group. SDG 3 (‘good health) was most cited (n = 4,255 references, 69.8%). Its frequency decreased significantly as country group GDP decreased. SDG 17 (‘partnerships’) became increasingly frequent as country income decreased, suggesting the fundamental role of partnerships in low-income countries. SDG 4 (‘education’) was mentioned more frequently in higher-income countries, but less in lower-income ones. Lastly, nutrition was mentioned more frequently in lower-income countries than higher-income ones, reflecting varying patient needs.

**Conclusions:**

This is the first bibliometric mapping study of SDGs in OFC care. The frequency of mentions of different SDGs varied by country income groups, suggesting different priorities and challenges to consider across global OFC research as we seek to achieve the SDGs by 2030.

The United Nations (UN) Sustainable Development Goals (SDGs) provide a globally recognised framework for addressing the interconnected social, economic, and environmental challenges that influence health and well-being. Adopted by all 193 UN member states at the 2015 UN Sustainable Development Summit, the 17 SDGs build upon the earlier Millennium Development Goals and establish a shared roadmap for global development through to 2030 [1]. Although SDG 3 (‘good health’) is most directly related to healthcare, many other goals influence health outcomes and health systems through their effects on education, inequality, economic opportunity, infrastructure, and environmental conditions. Good health, gender equality, availability of quality education, reduction of poverty, access to nutritious food and clean water, and the presence of strong infrastructures are all directly or indirectly interlinked with human health and the development of robust health systems [2], both in high-income countries (HICs) and low- and middle-income countries (LMICs). As a result, the SDGs offer a useful framework for evaluating how healthcare research and practice contribute to broader societal objectives and sustainable development.

Morbidity and mortality due to congenital conditions are becoming increasingly important in terms of their impact on child health worldwide [3], as improvements in communicable disease control contribute to reduction of burden of disease [4]. Orofacial clefts (OFCs) are one of the most common congenital conditions [5]. They can affect feeding, breathing, speech, hearing, appearance, dentition, educational outcomes, and psychosocial well-being [6]. However, with appropriate, timely, highly multidisciplinary care and follow up into adulthood, babies with an OFC can grow up to be healthy, productive individuals. Thus, the broad cross-disciplinary and lifelong challenges of OFC care and research may serve as an indicator of patterns across paediatric healthcare. Indeed, the age at which babies undergo an OFC lip repair has been proposed as an important bellwether for global paediatric surgical services [7].

Therefore, we sough to map the mentions of the 17 SDGs in global OFC research publications, as well as variations in the frequency with which themes relevant to each SDG are mentioned. Recognising potential global variations in healthcare challenges and research priorities, we wanted to identify any differences between the most frequently referenced SDGs across different country income groups, as defined by the World Bank [8].

## METHODS

Our data source was the International Centre for the Study of Research (ICSR) Laboratory [9], an Elsevier databricks platform which enables analysis of Scopus, Elsevier’s abstract and citation database of publication metadata sourced from various publishers, including, but not limited to, Elsevier itself. We searched the ICSR Laboratory database using Python, version 3.9 (Python Foundation, Wilmington, Delaware, USA) for any article on OFC. Given the interconnectedness of the SDGs and the importance of One Health and Planetary Health, and to capture any publication on OFCs and analyse the breadth of publications containing SDG mentions, we used an intentionally broad search strategy based on the following keywords: orofacial cleft, cleft palate, cleft lip, cleft speech, velopharyngeal dysfunction, velopharyngeal incompetence, velopharyngeal insufficiency, pharyngoplasty, pharyngeal flap. This approach ensured that we did not inadvertently exclude work related to SDGs that may not immediately be regarded as ‘relevant’ to OFC research. The keyword search was repeated on PubMed and Google scholar to compare the total number of articles retrieved.

Although the SDGs were formally introduced in 2015 [1], the themes they covered likely to have been included in prior publications. Moreover, publications that address topics directly related to SDGs may not always explicitly mention the relevant SDG. Therefore, we used established Elsevier methodology [10,11] to infer SDGs from titles, abstracts, and keywords. The ICSR Laboratory platform included publications from 1842 up to 2024, meaning that the manuscripts analysed here spanned 182 years. This specific timeframe was determined by data availability: the data were retrieved in 2024 during the active period of the ICSR laboratory, while the baseline date (1842) marks the earliest available literature indexed in PubMed.

We set no date restriction on the articles, meaning that articles published before the establishment of the SDGs were included in the analysis, provided that they were part of the Elsevier and Scopus literature. We otherwise only included articles with abstracts published in English.

### Variables

We determined country income groups based upon the country of affiliation of all authors. This meant that an article with authors from more than one country income group would be represented in more than one country income group. However, the World Bank’s income groups were introduced only in 1987, and some author affiliations were not available from digitised early literature from the 19th century. This meant that some papers had to be excluded from further analysis if they could not be matched with one or more World bank country group affiliations, or if they had no author affiliations.

We identified the research output from authors in each country income group and calculated the frequency of references to each SDG as a percentage of the total number of publications within each of the four country income groups. This allowed us to study any variations in the degree to which each country income group mentioned each SDG. We compared all four country income groups based on the percentages of SDG mentions within each group, rather than absolute numbers, to account for the much larger volume of research output from HICs than from LICs. This enabled the ranking of SDGs in descending order of frequency for each country income group.

### Statistical manipulation and data presentation

We used Spearman’s rank correlation to explore any differences in prevalence of SDG mentions by country income group. The figure presented in the manuscript was created on the Canva platform [12]. We report our findings per the STROBE guidelines [13]. The analysis was done in jamovi, version 2.6 (jamovi project, Sydney, Australia).

## RESULTS

Our keyword search retrieved 39,176 articles from the ICSR Laboratory, compared to 39,803 found on PubMed and 5410 on Google Scholar. We studied only the papers retrieved on ICSR Laboratory platform.

Of 39,176 the OFC-related articles, 5,598 (14%) mentioned SDG-related themes. The SDGs were individually mentioned 6,069 times, meaning that some articles mentioned more than one SDG theme. Therefore, 5,598 (14%) articles were mapped to at least one SDG-related theme. Of these 5,598 papers, 4,896 (87%) could be matched to an income country group, as some did not have an affiliation which the authors could retrieve.

All 17 SDGs were mentioned in our sample, albeit with wide variations in frequency (**Table 1**). Overall, SDG 3 was the most mentioned SDG, followed by SDG 17 (‘partnerships’) and SDG 4 (‘education’).

**Table 1:** Frequency of mentions of each of the 17 SDG themes across 5598 publications

SDG number and name	Number (%) of mentions
#3 – health	4,255 (69.80)
#17 – partnerships	1,078 (17.83)
#4 – education	211 (3.46)
#10 – reduced inequalities	148 (2.43)
#2 – nutrition (zero hunger)	105 (1.72)
#16 – peace, justice and strong institutions	45 (0.74)
#9 – infrastructure and innovation	39 (0.64)
#6 – clean water and sanitation	33 (0.54)
#11 – sustainable cities	29 (0.48)
#5 – gender equalities	29 (0.48)
#7 – affordable and clean energy	27 (0.44)
#1 – no poverty	23 (0.38)
#8 – economic growth and work	22 (0.36)
#15 – life on land	19 (0.31)
#14 – life below water	16 (0.26)
#12 – responsible consumption and production	14 (0.23)
#13 – climate action	3 (0.05)
Total	6,096 (100.00)

Our analysis further revealed SDG-related themes which may not seem directly related to OFC care, such as SDG 14 (‘life below water’). To validate our code and check for possible errors or misinterpretation, one author (ACC) reviewed the related articles manually. They discussed topics such as OFCs in animals such as seals [14] or environmental pollutants and their impact on the developments of OFCs in intrauterine gestation [15].

We noted a marked decrease in research output as country income status decreased. Therefore, to enable meaningful comparisons across country income groups, we report findings for each group’s relative percentages, as well as raw frequencies (**Table 2**). Reflecting findings in the total number of publications, SDG 3 was the most frequently mentioned SDG in articles whose authors were based in HICs, UMICs and LMICs. However, SDG 3 was no longer the most frequently referenced SDG in LICs, where SDG 17 emerged as the most frequent. Additionally, the relative percentage of mentions of SDG 3 progressively decreases as country income status decreases (*P* < 0.001). In contrast, there was no statistically significant association (*P* = 0.078) between the percentage of SDG 17 mentions and country income status (**Table 2**).

**Table 2:** Five most frequently mentioned SDGs for each country income group

Country income group (number of affiliations)	Most frequent SDGs	Number of affiliations (%)
HIC (3,717)	#3 – health	2,753 (74.00)
	#17 – partnerships	434 (11.70)
	#4 – education	170 (4.60)
	#10 – reduced inequalities	104 (2.80)
	#2 – nutrition (zero hunger)	54 (1.45)
UMIC (1,336)	#3 – health	755 (56.50)
	#17 – partnerships	440 (32.90)
	#4 – education	28 (2.10)
	#10 – reduced inequalities	25 (1.87)
	#2 – nutrition (zero hunger)	23 (1.72)
LMIC (772)	#3 – health	755 (56.50)
	#17 – partnerships	440 (32.90)
	#4 – education	28 (2.10)
	#10 – reduced inequalities	25 (1.87)
	#2 – nutrition (zero hunger)	23 (1.72)
LIC (166)	#17 – partnerships	68 (41.00)
	#3 – health	62 (37.30)
	#2 – nutrition (zero hunger)	15 (9.00)
	#10 – reduced inequalities	9 (5.42)
	#4 – education	3 (1.81)

HIC – high-income country, LIC – low-income countries, LMIC – lower-middle-income countries, UMIC – upper-middle-income countries

**Figure 1:**
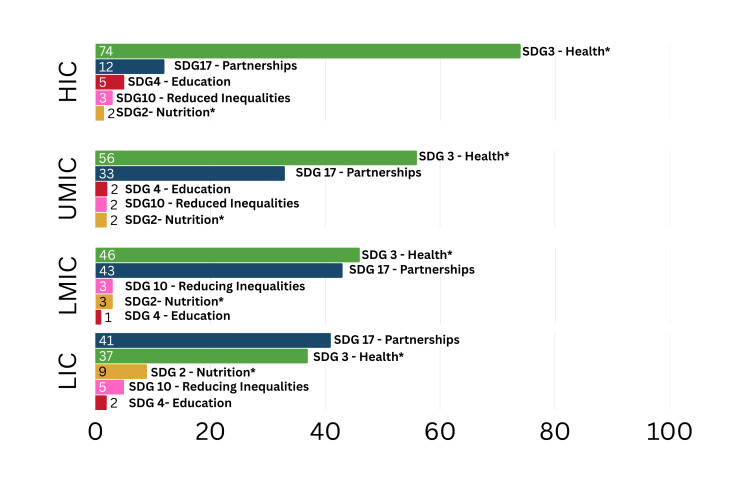
Five most frequently mentioned SDGs for each country income group expressed as a percentage of all authors for each country income group. HIC – high-income country, LIC – low-income countries, LMIC – lower-middle-income countries, UMIC – upper-middle-income countries.

We observed a large difference in percentages between the SDG 3 (74%) and SDG 17 (12%) in HICs (**Figure 1**); and the progressive decrease in this difference between these two SDGs, as country income status decreases, culminating in a reversal of positions of SDG 17 and SDG 3 in LICs.

In all four country income groups, the third, fourth, and fifth most frequently mentioned SDG accounted for a small percentage (1–9%) of SDG mentions. The SDG 2 (‘nutrition’) was the third most frequently mentioned SDG (9%) in LIC-based articles, but did not feature in the top three most frequently mentioned SDGs in any other country income group, ranking fourth in LMICs and fifth in both HICs and UMICs. Analysis by individual countries showed that the frequency of mentions of SDG 2 had a negative correlation with GDP (*P* < 0.001).

Additionally, comparing only the third-ranked SDGs across all four country groups, SDG 2 was mentioned by LIC-based articles, more frequently than the third ‘ranked’ SDGs in any other country group, *i.e.* SDG 4 in HICs and UMICs, and SDG 10 (‘reducing inequalities’) in LMICs.

## DISCUSSION

Only 14% of papers in this study were mapped directly to one of the SDGs. While SDG 3 is the most frequent (4,255 mentions), one may wonder why all articles ever written about cleft are not coded as discussing the theme of SDG 3. The reason for this is because of what the UN describes as being part of ‘good health’ – as explained in the paragraph, this corresponds to: SDG 3.2 (‘ending preventable deaths of children and newborns’) and SDG 3.4 (‘reducing premature mortality from non-communicable diseases’) [16]. The SDG 3.8 (‘universal health coverage’) and SDG 3.d (‘risk management and reduction of national and global health risks’) are also included in the definition of SDG 3 [17]. Thus, all health research topics may not map directly to SDG 3 subthemes prioritised by the UN. This raises the question of how effectively OFC research is addressing key global priorities and may help to inform more equitable and impactful allocation of research resources.

The findings in our large dataset align with those of other research published after the completion of our paper, where Mustakim *et al*. [18] manually evaluated a smaller selection of articles. Despite methodological differences, Mustakim *et al*. [18] and ourselves identified SDG 3, SDG 17, and SDG 4 as the dominant themes in OFC sustainability-related literature, supporting consistency across different analytical approaches. Indeed, in our analysis of 6,096 mentions, SDG 3 accounted for 4,255 (69.8%) of all SDG mentions, SDG 17 for 1,078 (17.8%), and SDG 4 for 211 (3.5%). Mustakim *et al*. [18] also emphasises the importance of SDG 2 and SDG 1 (‘no poverty’) in low resource environments where these might hinder OFC care and overall well-being of the local population. However, in contrast to a manual review, SDG mapping [19] enables searching large data sets for bibliometric exploration. Previous literature has compared Elsevier's script with other SGD mapping tools, and while none have the same accuracy as manual searching, the Elsevier tool was found to be slightly more stable than others [10].

Unsurprisingly, SDG 3 was the most mentioned SDG in the OFC literature, followed by SDG 17; however, the frequency of mentions of SDG 3 progressively decreased with country income status (*P* < 0.001), so much so that it became the second most frequently mentioned SDG in LICs, with SDG 17 taking its place.

Partnerships are fundamental for global healthcare and research. However, we observed no significant association between SDG 17 and country income (*P* = 0.078). A potential explanation is that in LMICs, foundations and non-governmental organisations have played, and continue to play, a large role in many aspects of specialist or multidisciplinary care for congenital conditions such as OFC [20]. For this reason, when looking at the different definitions of SDG 17, it is apparent how connected these are with the topic of OFC care. SDG 17.6, for example, promotes cooperation at different geographic levels to enhance knowledge, as well as access to science, technology and innovation, while SDG 17.9 encourages the implementation of international cooperation to achieve the SDGs [21].

Importantly, while our study clearly illustrates the greater focus on SDG 17 (‘partnerships’) in articles from lower resource settings, our findings represent only a reference to, or the existence of, a partnership, irrespective of its quality, effectiveness or sustainability. The proportionately much greater role and impact of partnerships in LICs, highlights the importance of robust, reproducible metrics to evaluate their effectiveness and sustainability, in particular, capacity building for healthcare and research.

The variation in the distribution of frequency of mentions of SDGs from HIC to LIC is striking. In HICs, publications mention SDG 3 far more frequently (n = 2753, 74%) than all 16 other SDGs combined (n = 964, 26%). In contrast in LICs, SDG 3 remains important (n = 62, 37%), but it cannot be taken in isolation without the contribution (n = 68, 41%) of SDG 17. The wider spread of SDGs addressed in articles from LICs suggests that a wider range of economic, development, infrastructure, and health system variables play a role in healthcare, and that focusing solely on direct medical or surgical interventions in LIC settings is not the only priority.

The SDG4 (‘supporting educational outcomes’) is particularly important given the various ways in which an OFC can adversely impact education. Children with untreated or poorly treated OFCs can face significant communication challenges due to speech and hearing difficulties [22], and some have specific learning difficulties. Further, children with visible differences often face stigma and bullying in communities and schools [23]. These difficulties can directly impact learning outcomes, including reading and comprehension skills, highlighting the need for specific learning support for children affected by orofacial clefts. However, the heading ‘education’ is not only linked with primary and secondary educational opportunities for children affected by congenital conditions when it comes to SDG definitions. It also included articles about surgical education for healthcare professionals, including the use of virtual resources and increasing the confidence of healthcare professionals in the field of OFCs. Indeed, while SGD 4.1 encourages to strive for quality primary education for all children, including those affected by health difficulties, SDG 4.4 promotes education for youth and adults, for them to have the relevant skills necessary for specific employment and jobs [21]. On one hand, this subtopic could be interpreted to include continuing professional education for healthcare professionals. However, it could be argued that work under the theme of health professional education would be better represented by SDG 3.C, which refers to health work force planning, including recruitment, development, and training [21]. The code we used to classify how SDGs also retrieved educational papers about surgical simulation related to OFC. While it is understandable how such terms would be included under SDG 4, there is a strong argument that these should strictly fall under SDG 3.C (‘healthcare professionals’ education’). Indeed, the use of simulation technology which enhances health education is potentially an important global development to democratise access to health training worldwide. If the SDGs were to be reviewed by 2030, the year by which the global community is encouraged to achieve them, there may be an opportunity to bring some clarity around topics which might have emerged post-establishment of the SDGs in 2015. Simulation and virtual education, for example, might fall under this category.

Notably, SDG 4 was the third most frequently mentioned goal in HICs and UMICs, compared to the fifth most frequently mentioned goal in LMICs and LICs. This suggests a different hierarchy of needs in healthcare across country income groups [24]. Indeed, SDG 4 loses its higher position in LMIC and LIC, where instead SDG 2 and SDG 10 are more prominent. This indicates that LMICs and LICs show different priorities compared to their counterparts, including reduced inequalities and nutrition. This underscores the different challenges that patients, researchers, and healthcare workers might face depending on the economic status of their country and institutions. While our study focuses on OFC literature, it serves as a potential indicator of similar challenges in other areas of global health.

Although the numbers of authors from LICs is small in our sample, it such contexts, SDG 4 was followed by SDG 15 (‘life on land’) in terms of frequency, as both were represented by the same number of articles (n = 3). A manual review of the SDG 15 articles showed they are related to chimpanzees’ and gorillas’ health in the context of OFCs. While animal OFC research is an important topic, it can be argued that educational outcomes in patients affected by OFC is of greater importance and societal impact. Our study highlights the lack of attention this important topic has received in OFC research to date, strongly suggesting that additional resources and funding are needed in the field of education for children affected by congenital conditions in LICs.

In relation to SDG 2, its frequency of mentions is inversely proportional to GDP or income country groups, meaning it increases as the country group’s GDP decreases (*P* < 0.001). A scoping review found that children born with cleft palates in Africa are twice as likely to be malnourished compared to their peers [25]. In LMICs, lack of feeding support and advice leads to preventable morbidity and mortality and increases the risk of any surgical intervention. Specific nutritional programmes could support children and their families in these contexts [26]. This issue could be less pressing in HICs, where children could be more likely to receive OFC specialist care earlier when it comes to nutritional support, as well as surgical interventions. This connects well with SDG 2.1 and SDG 2.2, which support safe and nutritious food for all people, as well as ending malnutrition. Apart from being related to OFC care, these goals apply more broadly to global health and surgery; according to the UNICEF, about half of deaths of patients under 5 is related to undernutrition or malnutrition [27]. Assessing and improving this would improve care for not only for babies with an OFC and related congenital anomalies, but for all children affected by low access to nutritious food.

The SDG 10 appears as the third or fourth most mentioned SDG in all country income groups, with no significant difference in prevalence of mention across country groups (*P* = 0.29). The SDG 10.2 encourages the social, political, and economic inclusion of all people, including those with disabilities [18]. Visible facial differences and speech difficulties in patients with OFC can be associated with stigma which is a barrier to social inclusion. Although the numbers of authors mentioning SDG 10 was relatively small compared with the other SDGs, this suggests that all country income groups face challenges with addressing inequalities.

### Generalisability

The proposal and methodology behind this article can be applied across a range of congenital and acquired diseases, particularly those that require lifelong multidisciplinary care. For instance, burden-heavy paediatric presentations such as cardiovascular diseases or oncological presentations could be examined following a similar methodology, thus broadening the perspectives on the intersection between SDGs and health interventions globally.

### Strengths and limitations

Our study includes papers published before the SDGs were developed and disseminated. Utilising the code developed by ICSR Laboratory [9] was important in our attempts to ensure the focus on SDGs in the OFC literature was represented as accurately as possible, even if the authors had not explicitly mentioned the SDG by name. This was important for two reasons. First, prior to the publication and dissemination of awareness of the SDGs, authors may have worked on relevant topics, but would not have directly mentioned relevant SDGs. Second, awareness of SDGs and their importance is not universal, even after their publication in 2015, and authors may address relevant, topics but not specifically mention the corresponding SDG. Thus, we were able to analyse a large number of articles to identify differences between each of the four country income groups’ SDG research related outputs.

Despite our wide search strategy, only 14% of papers were coded as mentioning one or more SDGs. Importantly, there is a wide gap in the volume of research output from HICs compared to LICs, despite most of the burden of disease being borne by the latter countries. Our approach of comparing country income groups based on each groups’ relative percentages of SDG mentions is important for ensuring that global findings are not skewed by the high-volume output of HICs. However, an important caveat is, with only 166 articles from LICs, small changes in absolute numbers can have a very marked impact on relative percentages. Nonetheless, such comparative data provide potential insights into how research publications highlight global differences in healthcare challenges and priorities. Although we cannot comment on factors leading to these publications ,which could include health-system needs, availability and conditions of research funding, co-authorship structures, or indexing artefacts, our study gives an indication of how research resources have been allocated; such data are important to guide future research direction and funding if we are to achieve the SDGs by 2030.

Importantly, this work represents a mapping exercise using inferred SDG classifications from titles, abstracts, and keywords. It does not establish causation, quality of implementation, or the effectiveness of sustainability interventions. Our analysis was based on inferred SDGs, author and country data, as well as title, abstract, and keywords searches from a large dataset, in contrast to literature reviews focusing on analysis of full text. We acknowledge that there may be limitations in inferring SDGs using Elsevier's algorithm; and that human review of each of the articles’ full text may provide additional clarity and detail. To this point, we only wanted to determine whether a research publication mentioned one or more SDGs and did not measure whether the research focused on an intervention(s) to achieve the SDGs, or whether it studied challenges or failures in achieving SDGs. As awareness and recognition of the importance of SDGs improves, we hope that prospective recording of how published research addresses SDGs will become routine practice, thereby making it possible to reproduce our study in the future and thus evaluate changes over time.

Another limitation is our inclusion of articles with abstracts in English only, including articles that might have abstracts both in English and in a second language. Future research and similar algorithms should be built to analyse all research, regardless of the language in which it has been published. Further, we only analysed papers retrieved by on the ICSR Laboratory platform. However, a similar total number of articles were found on PubMed.

Lastly, an argument could be made that countries grouped in the same country-income group are incredibly diverse, as are their patient populations, cultures, health systems, and, therefore, research priorities. Hence, the nuance of the diversity between countries with similar GDPs *per capita* could be lost when making country income group comparisons. Future research could focus on different income groups, but attempt to integrate countries’ individuality to better represent this diversity.

## CONCLUSIONS

This is the first bibliometric mapping study of SDGs in OFC research. Unsurprisingly, SDG 3 was the most mentioned SDG. We, however, observed differences in the frequency of SDG mentions across different country income groups. While we cannot attribute reasons for these differences, the variation in the prevalence of SDG mentions suggests that SDG 17 and SDG 2 assume greater importance in countries with lower income. We found no significant difference between the prevalence of mentions of SDG 10 across country income groups. With increasing awareness of and funding to achieve the SDGs, this study will serve as a useful baseline against which to evaluate future changes in SDG mentions in the OFC literature.

## Data Availability

**Data availability:** This project studied metadata from the ICSR Laboratory. The data supporting the findings of this study are openly available in the Elsevier Data Repository at https://doi.org/10.17632/9sxdykm8s4.1. The Repository hosts the United Nations SDGs query methodology developed by Rivest *et al.* [11].
